# Assessment of Knowledge, Practice, Perception, and Expectations of Artificial Intelligence in Medical Care among Staff of a Tertiary Hospital

**DOI:** 10.4314/ejhs.v34i4.7

**Published:** 2024-07

**Authors:** Aondona David Daniel, Akwaras Nndunno Asheku, Yohanna Stephen, Gyuse Ngueikyor Abraham, De-kaa Niongun Lawrence Paul, Laadi Terrumun Swende, Ornguga Bamidele Ohiozoje, Grace Nwunuji Rimamnunra, Ngbede Matthew Ocheifa

**Affiliations:** 1 Department of Family Medicine. Federal Medical Centre Makurdi. Benue State. Nigeria; 2 Bingham University Teaching Hospital, Jos. Plateau State. Nigeria; 3 University of Calabar Teaching Hospital, Calabar, Cross River State, Nigeria; 4 Department of Epidemiology and Community Health, College of Health Sciences, Benue State University Makurdi, Benue State, Nigeria

**Keywords:** Artificial intelligence, expectation, knowledge, perception, practice

## Abstract

**Background:**

Artificial intelligence (AI) is the simulation of human intelligence processes by machines, especially computer systems. AI technology has wide applications in biomedicine and has real practical benefits in many medical applications. The aim was to assess the knowledge, practice, perception, and expectations about AI technology among staff of Federal Medical Centre Makurdi Benue state, Nigeria.

**Methods:**

This was a descriptive cross-sectional study over a period of three months from March to May 2023. The respondents were 18 years and above. The questionnaire was self-administered employing convenience sampling method to recruit responders. Data analysis was done using SPSS version 20.

**Result:**

A total of 384 respondents were recruited. The mean (SD) age of the respondents was 42.3(±11.1). Most were aged 41-50 (34.4%). There were more females (56% (215)). Most of the respondents (69% (264)) attested to knowing AI technology. However, the majority (87% (231)) of the 264 respondents who knew about AI technology did not have in-depth knowledge. Regarding practices, more than half of the respondents (55.3%) did not think AI makes their task easy. The majority of the respondents (90.3%) believed AI technology is essential in the medical field and most of the respondents (12.2%) were expecting to acquire AI technology skills in the future.

**Conclusion:**

The in-depth knowledge of AI technology was low. Most of the staff thought that AI technology did not make their task easy although they believe AI is essential in medical field and they expect the acquisition of more skills on AI technology in future.

## Introduction

A computer or a machine that does activities that ordinarily require human intellect such as speech recognition, visual perception, decision making and language translation is said to demonstrate Artificial Intelligence (AI) capabilities (1). The integration of AI, a cutting edge technology to health care services has completely revolutionized healthcare delivery (1,2). This has innovatively improved patient care with reported advantages as in prediction of disease prognosis and response to treatment(3). Physicians in future would have to accommodate AI and other digital applications that save time and effort in the management of diseases (4–6).

The knowledge of AI in the developing countries is still rudimentary (7). Generally, there is an atmosphere of foreignness regarding AI technology amongst medical staff and the need for educating healthcare workers especially in Nigeria about AI technology is pertinent (4,7). The perception of healthcare workers in most developed countries is that AI would soon replace some specialties like radiology and imaging (2,4). Most of the health care workers in Nigeria are not certain of the advantages of AI technology in the healthcare industry (7).

This study was carried out to assess the knowledge, practice, perception and expectation of AI among staff of the Federal Medical Centre Makurdi, a tertiary health institution located in Benue State Nigeria. The study identified the extent of knowledge, sought for the reason why AI practices are not common and identified perceptions that need to be encouraged or discouraged. The expectations of medical staff regarding AI technology were also assessed. This unraveled areas in the developing world that pose a formidable barrier to the acceptability of AI technology in the health care industry.

## Material and Methods

The Federal Medical Centre, Makurdi is a 450 bedded facility operating five sites in Makurdi town, the capital of Benue State. The Mission ward in Wadata, the permanent site at Apir, the Dento-Psychiatric Complex by Aper Aku stadium, the National Health Insurance Authority (NHIA) beside River Benue and the Federal Staff clinic. The estimated staff strength of the hospital is 2500. The study was a descriptive cross-sectional study of staff at Federal Medical Centre, Makurdi, Benue State from March to May 2023.

The Cochrane formular was employed to determine the minimum sample size for the study. The minimum sample size (N) calculated was 384 at 50% proportion

**Study population**: The inclusion criteria were all cadres of staff which include medical doctors, nurses, pharmacist, non-clinical staff and allied health staff (community health workers, biomedical engineers, ward attendants, health information technologists, environmental health officers) working at Federal Medical Centre, Makurdi. Those who were not at their place of work and those who were attending to critically ill patient at the time of the study were excluded from the study. A convenience sample technique was employed to recruit 384 participants from all cadre of staff.

### Study protocol

A pre-test was carried out at the General Out-Patient Clinic (GOPC of the NHIA) of Federal Medical Centre Makurdi which is located at a far distance (40km) away from Apir permanent center to prevent information bias. The purpose of the study was explained to the staff and those who consented showed their approval by appending their signature on the consent form. The clarity of the questions and the time required to fill the questionnaire was assessed.

A structured questionnaire modified from questions obtained from a Pakistan study that had been validated was employed (8). The questions were phrased and further checked and validated by a senior researcher to be more appropriate for the respondents to answer comprehensively. The questionnaire was interviewer administered and obtained respondents' socio-demographic data, relevant history, knowledge of AI, perception and expectations of staff on the use or application of AI. Regarding the assessment of knowledge of AI, those who knew about Deep Learning (DL) and Machine Learning (ML) were considered to have good knowledge of AI and vice versa.

**Data analysis**: Data was analyzed using SPSS version 20. Descriptive statistics was performed with the level of significance set at p < 0.05.

**Ethical approval**: The ethical approval was obtained from the Ethical and Health Research Committee of Federal Medical Center Makurdi. The study was done in accordance with acceptable ethical standards as stipulated in the Helsinki Declaration of the 1975 and revised in 1983.

## Results

Table 1 reveals that most of respondents (34.4%) were within the age group 41-50 years. More than half were female and married and one-half (50%) had attained postgraduate level of education. More than half of the respondents (59.1%) were allied health workers and most (53.9%) had a monthly income above ₦100,000.

**Table 1:** Socio-demographic characteristics of respondents (n=384)

Socio-demographic characteristics	Frequency (%)
**Age (years)**..	
≤ 20	5(1.3)
21-30	50(13.0)
31-40	123(32.0)
41-50	132(34.4)
51-60	41(10.7)
≥61	33(8.6)
	
**Gender**	
Male	169(46.0)
Female	215(56.0)
**Marital status**	
Single	75(19.5)
Married	227(59.1)
Divorce/Separated	37(9.6)
Widow/Widower	45(11.7)
**Ethnic group**	
Tiv	151(39.3)
Idoma	122(71.1)
Igede	72(18.8)
Others	39(10.2)
**Religion**	
Christian	356(92.7)
Muslim	10(2.6)
Traditional	18(4.7)
**Level of education**	
Secondary	12(3.1)
Tertiary	180(46.9)
Postgraduate	192(50.0)
**Health Discipline**	
Doctors	45(11.7)
Nurses	13(3.4)
Pharmacist	10(2.6)
Allied health workers	227(59.1)
Non clinical staffs	89(23.2)
**Monthly income**	
<30,000	26(6.8)
30000 - 99000	151(39.3)
100000 and above	207(53.9)

Table 2 shows that only about a third (34.4%) of the respondents browsed the internet on daily basis to seek for health-related information and most of the respondents (67.4%) had not used medical monitoring device personally or for a relative before.

**Table 2:** Relevant history related to AI

Variables	Frequency	Percentage
**Frequency of usage of internet to obtain health-related information**.		
Daily	132	34.4
Weekly	113	29.4
monthly	139	36.2
**Usage of medical monitoring device personally or for a relative**		
Yes	125	32.6
No	259	67.4

Only 69% of the respondents knew about AI.

Table 3 shows that the majority of the respondents (87%) did not have in-depth knowledge of AI (They did not know what Deep Learning DL and Machine Learning ML were). Most of the staff (98.8%) responded correctly that AI was the ability of a machine to function as a human in problem-solving. The majority (74.2%) had not read about AI in the past six months. A high percentage of the respondents (83%) knew about an application of AI in the medical field but most (84.5%) did not know any facility where AI is utilized.

**Table 3:** Knowledge of AI (n=264)

Variable	Frequency	Percentage
**Knowledge of deep learning (DL) and Machine learning (ML)**		
Yes (have in-depth knowledge)	33	12.5
No	231	87.5
**AI is the ability of a machine to function as human in problem solving**		
True (correct)	261	98.9
False	3	1.10
**Read about AI in the past six month**		
**Yes**	68	25.8
No	196	74.2
**knowledge about any application of AI in the medical field**		
Yes	219	83.0
No	45	17.0
**Knowledge of any facility where AI is utilized**		
Yes	41	15.5
No	223	84.5

Table 4 shows that more than one-half of the respondents (55.3%) did not think AI made their task easy and most of the respondents (61%) opined that it was not easy to apply AI technology. Although, majority of the respondents (83%) positively affirmed that they would want to work with AI in future.

**Table 4:** Practice of AI

Variable	Frequency (%)
**AI make your task easy**	
Yes	38(14.4)
No	146(55.3)
Not sure	80(30.3)
**Easy to apply AI**	
Yes	20(7.6)
No	161(61.0)
Not sure	83(31.4)
**Would like to work with AI in future**	
Yes	219(83.0)
No	45(17.0)

Table 5 shows that majority of the respondents (90.3%) believed that AI is essential in medical field and most (84.1%) of the respondents did not believe AI would replace the role of healthcare workers in future. A high percentage of the respondents (93.2%) thought the use of AI would give more time for healthcare workers to educate patients. More than one-half (58.3%) believed AI would have less treatment error in future. Most of the respondents (80.7%) thought the use of AI would reduce burn out syndrome in future and more than two-third (70.5%) did not believe AI would increase violation of patient confidentiality. Majority of the respondents (90.9%) believed budgets should be allocated for AI in the management of disease burden in Nigeria.

**Table 5:** Perception about AI

Variable	Frequency	Percentage
**AI is essential in medical field?**		
Yes	240	90.3
No	24	0.91
**AI would replace the role of healthcare workers in future**		
Yes	42	15.9
No	222	84.1
**AI would give more time for HCWs to educate patients**		
Yes	246	93.2
No	18	6.8
**AI would have less treatment error in future**		
Yes	154	58.3
No	110	41.7
**AI would reduce burnt out syndrome in future**		
Yes	213	80.7
No	51	19.3
**AI would increase violation of patient confidentiality**		
Yes	75	28.4
No	186	70.5
Not sure	3	1.1
**Budget should be allocated for AI in management of disease burden and pandemic in Nigeria**		
Yes	240	90.9
No	24	9.1

**Figure 2:**
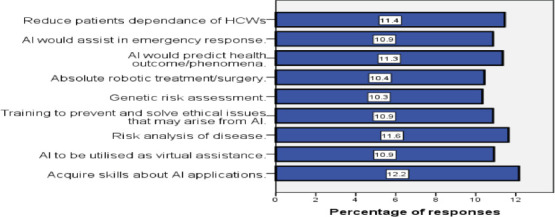
Expectation of Respondents to AI

Figure 2 is a bar chart that shows that most of the respondents (12.2%) were expecting to acquire skills in AI applications in future. Respondents who believed AI could be used for risk analysis were 11.6%, while those who believed AI would predict health outcomes were 11.3%. About 10.9% of the respondents believed that AI would assist in emergency response, could be utilized as virtual assistant, and expected that training of staff on AI technology would be essential to prevent and solve AI-related ethical issues.

## Discussion

This study assessed the knowledge, practice, perception and expectation of AI among staff of Federal Medical Centre, Makurdi. Most of the respondents were female, married and attained postgraduate level of education. Majority were in the allied health workers cadre and within the age group of 41-50 years. Studies on AI in other health related institutions in Nigeria, Ghana and Saudi Arabia revealed similar socio-demographic characteristics (1,3,7). This economically productive population group in the health sector are by the nature of their work exposed to technological innovations like AI.

The study revealed that only a third of the respondents browse the internet on daily basis for health-related issues and most of the respondents did not employ devices like digital blood pressure and thermometer monitoring their health status. There is a positive correlation between the use of internet for health related purposes, use of medical monitoring devices and acceptability of AI technology in health (10,11). The likelihood of low acceptability to innovative technology is imminent in this population group due to sophistication and myth surrounding internet network and unfamiliar medical devices though working in a health setting. The respondents that knew about AI in this study were 31% out of which only 12.5% had in-depth knowledge of AI. (knew about Deep Learning - DL and Machine Learning - ML). A higher proportion of knowledge about AI has been reported in several studies. The study in a health institution in France revealed that 90% knew what AI was and 40% demonstrated in-depth knowledge (12). In Pakistan, a study revealed that 71.3% knew about AI technology in a health setting but only 35.3% had in-depth knowledge (8). The proportion of those with knowledge of AI in the United States of America was 65.9% of which 42.3% had indepth knowledge (13). The low proportion in this study could be due to the rudimentary knowledge of AI in developing countries like Africa and the uncertainty of the advantage of AI technology in the healthcare setting.

The staff in this study attested that it was not easy to apply AI technology in their practice though they would want to include AI in their practice in future. Several studies in Nigeria, Pakistan, Saudi Arabia and North-Eastern Spain have also opined difficulties in working with AI technology (8,11,14,15). In Africa, the low level of digital literacy, low internet penetration and inaccessibility to electricity has hampered seamless operation of AI technology.

Most of the staff in this study perceived that AI technology was essential in medical field and would not replace the role of health care workers. The staff also believed AI technology would give more time for health care workers to educate their patients about the conditions being managed. Alowaise et al in Saudi Arabia also emphasized the perceived significance of AI technology and its benefit in the medical field (16). Studies in Nigeria, Paris and India had also highlighted the perceived advantage of gaining time to interact with patients and preventing burn out syndrome with the introduction of AI technology in the medical field (14,17,18)

The expectation of most respondents in this study was to acquire skills about AI in future. Assistance in emergency response, training of staff on AI technology, risk analysis and prediction of health outcomes were also some of the expectations advanced. Owoyemi et al and Ade-Ibijola in Nigeria also documented the optimism of respondents embracing AI technology in health institutions in their study (7,14). Park et al in South Korea and Davenport et al in the United State of America also affirmed that the expectation of their respondents mostly was that AI technology would bring innovation to the existing medical technologies in the future(19,20).

This cross-sectional study may not represent the situation at another time. The association between variables may be difficult to determine due to limitations in controlling all possible confounders.

In conclusion, most staff had limited in-depth knowledge and application of AI technology in their practice. There is a strong perception of AI's importance in medicine and an expectation to acquire AI skills. Policies and training to enhance AI knowledge, utilization, and improve infrastructure are needed in developing countries like Nigeria.
